# Causes and outcomes of at-risk underperforming pharmacy students: implications for policy and practice

**DOI:** 10.1186/s12909-024-05327-z

**Published:** 2024-04-19

**Authors:** Alice Campbell, Tina Hinton, Narelle C. da Costa, Sian E. O’Brian, Danielle R. Liang, Nial J. Wheate

**Affiliations:** 1 Sydney Pharmacy School, Faculty of Medicine and Health, The University of Sydney, 2006 Sydney, NSW Australia; 2 Charles Perkin Centre, Faculty of Medicine and Health, The University of Sydney NSW, 2006 Sydney, NSW Australia

**Keywords:** Higher education, University, Underperformance, Pharmacy, Show cause, At risk

## Abstract

**Objective:**

This study aimed to understand the key determinants for poor academic performance of students completing a Bachelor of Pharmacy (BPharm), Bachelor of Pharmacy and Management (BPharmMgmt), or Master of Pharmacy (MPharm) degree.

**Methods:**

Data were collected on pharmacy students who had not met academic progression requirements between 2008 and 2018 at The University of Sydney, Australia. This included: age at the start of pharmacy degree; gender; whether they transferred from another university; whether they were a domestic or international student; Australian Tertiary Admissions Rank upon entry, previous studies in biology, chemistry, or mathematics; show cause triggers (units of study failed); number of show causes; students’ written show cause responses; weighted average mark at last show cause or graduation; whether they graduated and were a registered pharmacist; and, the number of years they spent studying the degree. Descriptive studies were used to analyse student characteristics using SPSS software, and student self-reported reasons for poor performance were analysed reflexively using thematic analysis procedures using NVivo.

**Results:**

This study included 164 pharmacy students enrolled in a BPharm (79.3%, *n* = 130), BPharmMgmt (1.2%, *n* = 2), or MPharm (19.5%, *n* = 32). Of the students, 54% (*n* = 88) were men, 81% (*n* = 133) were domestic students, 15% (*n* = 24) transferred from another degree program, and 38% (*n* = 62) graduated from the course. Show cause students were less likely to graduate if they transferred from another degree program (*P* = 0.0002) or failed more than three units of study (UoS; *P* < 0.0001). The most commonly failed UoS were related to organic or pharmaceutical chemistry, and the top student self-reported reasons for poor performance was stress/anxiety, physical health, and depression.

**Conclusion:**

Pharmacy schools should aim to address student foundational knowledge in chemistry, identify at-risk students early using pre-subject testing, and provide better services to address student mental health.

**Supplementary Information:**

The online version contains supplementary material available at 10.1186/s12909-024-05327-z.

## Introduction

A student’s academic performance in higher education is typically defined by their achievement of learning outcomes and demonstration of their ability to apply the concepts taught. Measurement of these attributes can include assessments, quizzes, role plays, field work, practical placements, workshops, tutorials, laboratories, and examinations. In most higher education programs, a minimum standard of academic achievement is required in order to progress through the course, to ensure the student has gained adequate knowledge and skills, and that they have achieved the specified learning outcomes. In this regard, poor academic performance can be defined by instances where a student fails to meet the expected minimum academic standard. Usually this comprises a minimum overall score in a subject and/or passing a specific barrier assessment, which is ultimately linked to their retention or attrition.

Understanding the key determinants of student success, failure, retention, and attrition has become increasingly important for higher education institutions, and has been the subject of extensive research over the past few decades. Early studies on student attrition focused primarily on student characteristics [1], before attention shifted to interactions between the student and their institutions. Prominent researchers, including Spady [2, 3], Tinto [4, 5], and Bean [6] proposed models to explain the interplay between academic and social integration leading to underperformance, and eventually, attrition. More recently, interest has increased in examining student engagement [7–9], where the student and institutions have a joint responsibility for academic success. To be successful, a student needs to participate, and higher education institutions need to provide an appropriate learning environment, opportunities, and support [10].

Studies on the key determinants of student underperformance reveal an array of contributing factors. Recent systematic reviews on underperformance and dropout rates show that key determinants fall into categories relating to the institution, personal life, demographics, and social integration [11, 12]. Within higher education institutions, studies have found that an academic’s professional knowledge and pedagogical skills, along with the institution’s learning resources, course structure, and environment, are key factors that influence academic performance and non-completion [13–17]. Teaching methods that higher institutions adopt have also been evaluated, with student-centered approaches that encourage active learning resulting in better performance when compared with a traditional teacher-centered approach [15, 16].

In terms of individual factors, studies have found a lack of effort, distraction, poor time management, and no longer being interested in the course as having a negative impact on academic performance [14, 15, 18, 19]. Active learning (e.g. self-quizzes, completing problem sets, and explaining concepts) has been found to yield better academic outcomes when compared with passive learning (e.g. reading lecture slides or class notes, watching lecture videos, and reading textbooks) [20, 21]. In the same study, how early a student studied in relation to their exam did not affect their outcome, whereas students who were more distracted during the time they allocated for study, performed worst [20, 22]. Education-related stress, poor mental health, exam anxiety, and sleep quality are also factors found to cause poor performance [23–27]. Other studies have shown that part-time students and those who have previously failed subjects are at risk of further poor performance and attrition [17, 28, 29]. Social factors including cyberbullying [30], homesickness for international students [31], and excessive socialising [16] also have a negative effect on academic performance.

Working status was found to negatively impact academic performance [27], where poor academic outcomes were correlated with a longer time spent at work [16, 28, 32]. Many studies have associated the lower socioeconomic status of students and their family, or financial strain with poor academic performance [27–29]; whereas, other studies have shown that students in families where one parent has attended higher education tend to achieve higher grades [31]. Some studies have found men and minority students are more at risk of poor performance [31, 33]. Part-time students are much more likely cite work and family responsibilities as reasons for stopping their studies [17]. Research on students whose first language is not that of the higher education institution is mixed, with some confirming it to be a key attributor to underperformance [34–36], along with students with a migrant background or who are first-generation university attendees (commonly referred to as first-in-family) [31, 37, 38]. In contrast, other studies have found that academic performance of international students was similar, or better, than domestic students [39, 40].

A government panel in Australia reported that the leading drivers for non-completion in higher education are both institution-related (learning environment, an academic’s ability to teach, student to staff ratios, student engagement, and support services) and student-related (health, finance, and personal responsibilities) [41]. A survey conducted by the Australian Bureau of Statistics (ABS) identified the top three reasons for attrition for students studying a bachelors degree to be: loss of interest, employment/financial reasons, and personal reasons (health, family, or other personal reasons). For postgraduate courses, reasons for attrition were highest in the order of personal reasons, employment/financial, followed by loss of interest [42].

Where a student has underperformed, they may be offered remediation assessments; to re-enroll and attempt the entire subject again, which may result in a delay in degree completion; or in some cases, be excluded from reenrolling into the same course for a period of time [43, 44].

Consequences of poor performance vary across higher education institutions and may depend on the reasoning provided, extent of underperformance, and number of failed subjects. Key stake holders impacted by poor performance and attrition from higher education can include the students and their families, the higher education institution they are enrolled in, their community workforce, and government. Non-completion directly impacts the funding and reputation of an institution [17, 45, 46]. In Australia, where the cost of higher education for domestic students is subsidised by the federal government, non-completion incurs a direct cost to both the student and the tax-payer. The cost to the student includes lost time, psychological health, student debt, and forgone income [9]. From the perspective of workforce planning, a delay or non-completion of study reduces the number of employees entering into the workforce, and can lead to workforce shortages and place a burden on those currently in the field.

There are many studies that have examined the key determinants for student success or underperformance and attrition in health; however, most have focused on nursing or medical education [13, 15, 47–50]. Consequently there are limited studies that have examined the rate and reasons for attrition within pharmacy degrees. Being a degree known to be difficult in technical content, and which requires students to achieve a high level of competence, it is important to investigate reasons for attrition and potential opportunities for improvement in student teaching and engagement.

In this study we analysed 10 years of demographic data and responses to why academic progression requirements had not been met in a cohort of students enrolled in a Bachelor of Pharmacy (BPharm), Bachelor of Pharmacy and Management (BPharmMgmt), or Master of Pharmacy (MPharm) degree at The University of Sydney. Our aim was to understand the key determinants for poor performance within this group of students and identify opportunities for policy and practice to reduce underperformance in the future.

## Methods

### Ethics

Approval for this study was granted by the Human Research Ethics Committee of The University of Sydney (2022/815).

### Data collection

The inclusion criteria for this study were students enrolled in a BPharm, BPharmMgmt, or MPharm degree between the period of 2008 and 2018 (inclusive), who were required to provide a minimum of one show cause at any stage of their study. Data collected on each student included: age at the start of pharmacy degree; gender; whether they transferred from another university; whether they were a domestic or international student; Australian Tertiary Admissions Rank (ATAR) upon entry, which is a percentile score that ranks Australian students finishing secondary school in relation to their academic achievement [51]; previous studies in biology, chemistry, or mathematics; show cause triggers (units of study failed); number of show causes; students’ written show cause responses; weighted average mark (WAM) at last show cause or graduation (WAM is an average grade score indicating a student’s overall academic performance over the course of their degree and is similar to a grade point average) [52]; whether they graduated; and, the number of years they spent studying the degree. Whether those students who had graduated were currently registered as a pharmacist in Australia was retrieved using the Australian Health Practitioner Regulation Agency online registry list [accessed in 2023].

### Data analysis

Researchers Da Costa, O’Brien, and Liang collected, screened, and de-identified the data, and researchers Campbell, Hinton, and Wheate analysed the data. Descriptive statistics, including mean ± SD, median, and frequencies (count and percentage) were calculated using Microsoft Excel. Mann-Whitney U tests were undertaken in GraphPad Prism 9.0 (GraphPad Software, Boston, MA, USA) to ascertain any differences between ATAR scores. Chi Square analyses were undertaken in GraphPad Prism 9.0 to compare categorical data including differences between men and women, domestic and international students, transferring and non-transferring students, and graduating and non-graduating students.

Written show cause responses were transcribed by Campbell and uploaded into NVivo (1.5.1) software (QSR International, Massachussets USA). The show cause responses were analysed reflexively using inductive thematic analysis procedures [53].This involved manually reviewing each show cause response to identify emerging themes relating to the reasons stated by the student for their poor performance. From the themes identified, a total of 43 codes were generated based on the ideas, trends, and content. Coding was conducted in a theory-driven manner, seeking to code information referencing the specific themes arising from the show cause response [53]. Themes were guided by the frequency of mention, and reported in the results if there was more than a single mention. The frequency of the subthemes was analysed to demonstrate the prevalence of stated factors that the student believed led to their poor performance.

### Show cause process

**Fig. 1:**
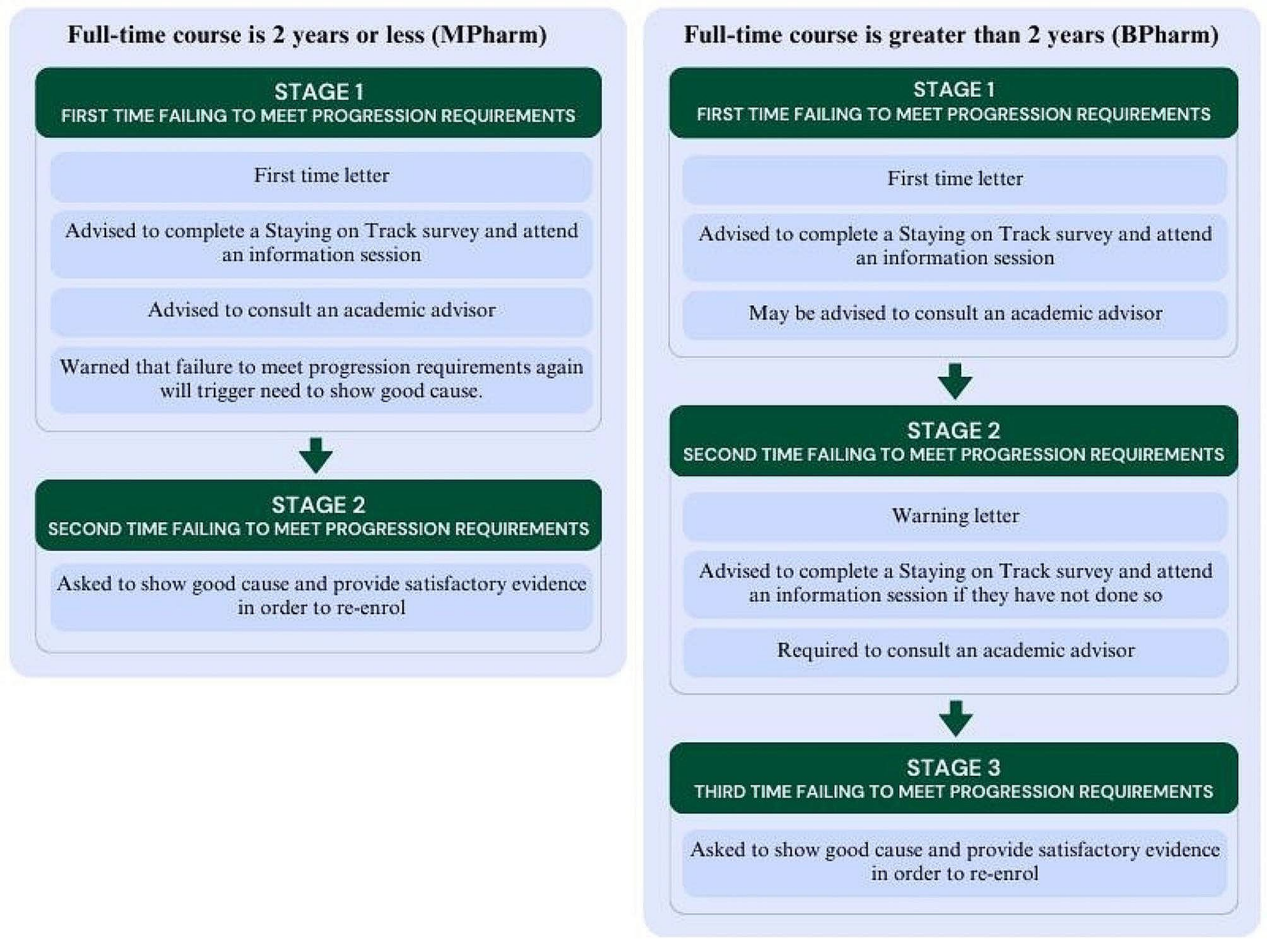
The three at-risk stages of academic intervention for students who fail to meet course progression requirements. Show cause is required at Stage 2 (MPharm) or Stage 3 (BPharm/BPharmMgmt) in order to re-enrol

Pharmacy students who do not meet the progression requirements of their degree enter one of three stages of academic intervention (Fig. 1). Triggers for a student not meeting the requirements for progression include: awarded a fail grade in over 50% of total units of study (subjects; UoS) taken in a semester or teaching period; an average grade (WAM) less than 50 across all UoS in a semester or teaching period; failing one, or more, barrier or compulsory UoS which includes CHEM1611, CHEM1612, PHAR2822, and any 3000 or 4000 level UoS for BPharm/BPharmMgmt; and any single UoS for MPharm; any practical component (e.g. field work or clinical work), failing the same UoS twice, having unsatisfactory attendance, or exceeding the maximum time limit allowed for the degree to be completed.

Students who fail to meet progression requirements for the first time are placed on Stage 1 of the at-risk register at which point they receive a letter from the Faculty of Medicine and Health, and are advised to complete a ‘Stay on Track’ survey and information session. At the discretion of the Associate Dean of Education, some students at Stage 1 may be required to consult an academic adviser. If a student is enrolled in a degree with a duration of less than two years full-time (e.g. MPharm), they are advised that should they fail to meet progression requirements in the following semester, they would be asked to ‘show good cause’ in order to be allowed to re-enrol in the same program; that is, they would be excluded from the degree for two years unless they could give reasons for why they should be allowed to remain studying. They are also recommended to speak to an academic advisor.

Stage 2 is triggered for a student in a 4 or 5 year undergraduate degree program (e.g. BPharm and BPharmMgmt) if they fail to meet progression requirements after being placed on Stage 1 in the previous semester, at which point the faculty sends a letter, advising the student to complete the ‘Staying on Track’ survey if they had not yet done so, and to consult an academic adviser. Stage 3 is triggered if a student fails to meet progression requirements a third time, or fails the same compulsory or barrier UoS, or any practical component twice. Students on Stage 3 are required to ‘show good cause’ and provide reasonable evidence to be allowed to re-enrol into the degree program.

## Results

### Demographics

In total, 164 pharmacy students received at least one show cause notification between the period of 2008 to 2018 (inclusive) and were enrolled in a BPharm (79.3%, *n* = 130), BPharmMgmt (1.2%, *n* = 2), or MPharm (19.5%, *n* = 32) degree (Table 1). Of the students, 54% (*n* = 88) were men, and 81% (*n* = 133) were domestic students.

Students who transferred from another degree program made up 15% (*n* = 24) of the sample, and were a median two years older than those who did not transfer (median age 21, range 19–43 years). All students who transferred from another degree, were enrolled in the BPharm. Ninety-two percent of transfer students (*n* = 22) were domestic and 71% (*n* = 17) were women.

The age of students at the start of their degree was positively skewed, with a median age of 19 years for BPharm and BPharmMgmt (range 17–43 years). For MPharm, the median age at commencement was 24 (range 20–24) years. The median age of domestic students at the start of their BPharm or BPharmMgmt degree was 19 (range 17–43) years compared with international students at 22 (range 18–33) years. For MPharm, the median age for domestic students at commencement was 24 (range 20–54) years while for international students it was 24.5 (range 22–38) years.

**Table 1:** Characteristics of the 164 pharmacy students required to show cause who were enrolled in either BPharm, BPharm/Mgmt, or MPharm degrees between 2008 to 2018

Characteristic	Value	
Age at start of degree (years), *median (min-max)*	19	[17–54]
Gender, *n* (%)		
Female	76	[46]
Male	88	[54]
Degree program, *n* (%)		
BPharm	130	(79)
BPharm/Mgmt	2	[1]
MPharm	32	[20]
ATAR*, *mean (min-max)*	88.8	(67.8–98.5)
Transferred from other degree program, *n (%)*		
No	140	(85)
Yes	24	[15]
Domestic or international, *n* (%)		
Domestic	133	(81)
International	31	[19]
Total UoS failed, *median (min-max)*		
BPharm	8	[2–33]
BPharm/Mgmt	9	[5–13]
MPharm	5	[2–12]
Number of show cause, *median (min-max)*		
BPharm	1	[1–8]
BPharm/Mgmt	2	[1–3]
MPharm	1	[1–4]
Graduated from degree program *n* (%)		
No	102	[62]
Yes	62	[38]
Time taken to graduate (years)*, *median (min-max)*		
BPharm	7	[4–10]
MPharm	3	(2.5–8)
Weighted Average Mark (WAM)*^, *median (min-max)*	52.7	(0–72.4)
Registered as pharmacist on AHPRA*, *n* (%)		
No	109	[66]
Yes	49	[30]
Unknown	6	[4]

*Missing values for each characteristic, where n represents the number of students without reported values: ATAR, *n* = 86; time taken to graduate, *n* = 102; WAM, *n* = 3; registered as Pharmacist on AHPRA, *n* = 6

^WAM at last show cause or at graduation

### Performance on entry and exit of the degree

The ATAR scores of the students in either the BPharm or BPharmMgmt were not normally distributed (*n* = 78, mean ATAR 88.8 ± 4.8) (Supplementary Figure S1). The average ATAR required for entry into BPharm and BPharm/Mgmt at the University of Sydney is around 90. Of the 24 students who transferred from another degree program, the ATAR score was available for four students, with an average of 78.8 ± 9.8, including two outliers who had ATAR scores of 67.80 and 74.15. The average ATAR on entry to the degree of the students who graduated was 89.4 ± 3.4, which was similar to those who did not graduate, 88.5 ± 5.4. A Mann-Whitney U test showed this difference was not statistically significant (W = 702.5, *p* = 0.937).

**Fig. 2:**
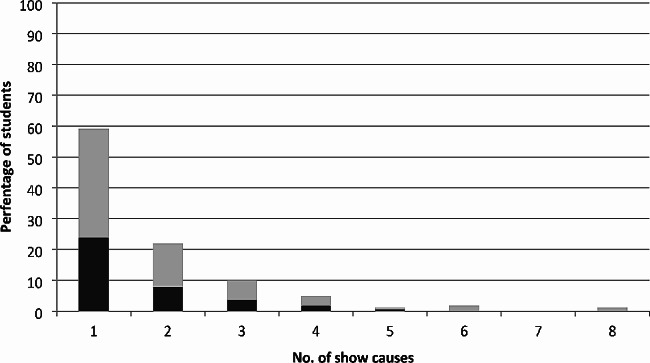
Percentage of students who graduated (black) and did not graduate (grey) by number of show causes received

The proportion of students who graduated after receiving at least one show cause was 37.8% (*n* = 62), of which 77.4% (*n* = 48) were registered as pharmacists at the time of data collection (Fig. 2). One student did not graduate their BPharm; however, they did return and complete the MPharm degree and was registered as a pharmacist at the time of data collection. The median time taken to graduation was 7 (range 1–9) years for students enrolled in the BPharm and 3 (range 2.5-8) years for those enrolled in the MPharm. During the study period, 188 students were enrolled in the BPharmMgmt degree but only two (1.1%) were required to show cause due to poor performance. Neither of those two students graduated.

A WAM score was available for all but three of the 164 students. The overall average WAM either at last show cause, if the student had not graduated, or at degree completion was 52.1 ± 12.0. For students who graduated (38.5%, *n* = 62), the average WAM was 62.2 ± 5.1, while for those who did not graduate (61.5%, *n* = 99), the average WAM was 45.7 ± 10.5.

When the proportion of students who graduated was compared across the ATAR bands (Table S1), it was evident that show cause students who entered their degree with an ATAR between 85 and 89.99 were more likely to graduate (44%) when compared with those who entered their degree with lower (27%) and higher (25–35%) ATAR scores.

### Units failed

Across the cohort, show cause students received between 1 and 8 show cause notifications (Fig. 1). When the proportion of students who graduated was compared across the number of show causes received for those who received 1–5 show causes, the rate of graduation ranged from 36 to 50%, while none of the students who received six or more show causes graduated.

### Number of failed UoS

The median number of UoS failed across the three degree programs was 8 (BPharm, range 2–33), 9 (BPharmMgmt, range 5–13), and 5 (MPharm, range 2–12), respectively. In total, 8.5% (*n* = 14) students were required to show cause because they failed 2 or 3 UoS, 19.5% (*n* = 32) students failed 4 or 5 UoS and 72% (*n* = 118) students failed more than 6 UoS. Of the 14 students who failed 2 or 3 UoS, 86% were studying the MPharm degree and the remaining were BPharm students. Students who failed 4 or 5 UoS, were studying a BPharm (66%), BPharmMgmt (3%), or MPharm (31%) degree. The majority of students who failed more than 6 units were studying BPharm (91%), followed by MPharm (8%), and BPharmMgmt (1%). Students who failed 2 or 3 UoS were significantly more likely to graduate when compared with those who failed 4 or 5 UoS, or more than 6 UoS $(X_2^2=21.86, \text{P}<0.0001)$ (Supplementary Figure S2).

### Type of failed UoS

The most failed UoS that triggered a show cause across students in the BPharm and BPharmMgmt degrees were a mix of pharmaceutical sciences, chemistry and biology, across the first and second years of the degree programs (Table 2). The top five UoS failed were Basic Pharmaceutical Sciences (8.8%, 116/1314 fails; unit code: PHAR1812), Chemistry 1B (Pharmacy) (6.9%, 91/1314 fails; unit code: CHEM1612), Drug Discovery and Design 1 (6.7%, 88/1465 fails; unit code: PHAR2811), Molecular Biology and Genetics (6.5%, 86/1314 fails; unit Code: MBLG1001), and Chemistry 1A (6.2%, 81/1314 fails; unit code: CHEM1611).

For students studying the MPharm, the majority of UoS failed were for pharmaceutical sciences in first year and one specific pharmacy practice unit (PHAR5717) in the second year. The top three UoS failed for MPharm were Pharmaceutical Chemistry 1A (12.6% 19/151 fails; unit code: PHAR5513), Pharmaceutical Science (7.9%, 12/151 fails; unit code: PHAR5515), and Pharmaceutical Chemistry 1B (7.9%, 12/151 fails; unit code: PHAR5516) (Table 3).

**Table 2:** Top 15 UoS failed in by BPharm and BPharmMgmt students

UoS Name	UoS Code	Category	Year	Degree	No. Failed
Basic Pharmaceutical Sciences	PHAR1812	PSCI	1st	B. Pharm	116
Chemistry 1B (Pharmacy)	CHEM1612	CHEM	1st	B. Pharm	91
				B. PharmMgt	
Drug Discovery and Design 1	PHAR2811	PSCI	2nd	B. Pharm	88
Molecular Biology and Genetics	MBLG1001	BIOL	1st	B. Pharm	86
Chemistry 1 A (Pharmacy)	CHEM1611	CHEM	1st	B. Pharm	81
				B. PharmMgt	
Drug Discovery and Design B	PHAR2821	PSCI	2nd	B. Pharm	60
Physical Pharmaceuticals and Formulations A	PHAR1822	PSCI	1st	B. Pharm	57
				B. PharmMgt	
Physiology for Pharmacy	PHSI2601	BIOL	2nd	B. Pharm	54
Microbiology and Infection	PHAR2812	PSCI	2nd	B. Pharm	53
Pharmacy Practice 1	PHAR1821	PRAC	1st	B. Pharm	51
				B. PharmMgt	
Foundations of Pharmacy	PHAR1811	PRAC	1st	B. Pharm	45
				B. PharmMgt	
Therapeutic Principles	PHAR2813	PSCI	2nd	B. Pharm	43
Physical Pharmaceutics and Formulation B	PHAR2823	PSCI	2nd	B. Pharm	41
Pharmacology for Pharmacy	PCOL2605	PCOL	2nd	B. Pharm	37
				B. PharmMgt	
Pharmacy Practice 2	PHAR2822	PRAC	2nd	B. Pharm	35
				B. PharmMgt	

PSCI = Pharmaceutical Sciences, CHEM = Chemistry, BIOL = Biology, PRAC = Pharmacy Practice, and PCOL = Pharmacology

**Table 3:** Top 9 UoS failed by MPharm students

UoS Name	UoS Code	Category	Year	Degree	No. Failed
Pharmaceutical Chemistry 1 A	PHAR5513	PSCI	1st	M. Pharm	19
Pharmaceutical Science	PHAR5515	PSCI	1st	M. Pharm	12
Pharmaceutical Chemistry 1B	PHAR5516	PSCI	1st	M. Pharm	12
Integrated Primary Health Care 2	PHAR5717	PRAC	2nd	M. Pharm	10
Pharmaceutics 1	PHAR5517	PSCI	1st	M. Pharm	9
Pharmacy Practice A	PHAR5518	PSCI	1st	M. Pharm	9
Medicinal and Pharmaceutical Chemistry	PHAR5713	PSCI	2nd	M. Pharm	9
Pharmaceutics 1	PHAR5514	PSCI	1st	M. Pharm	8
Pharmaceutical Microbiology	PHAR5712	PSCI	2nd	M. Pharm	8

### Gender, transfer and international students

There was no significant difference between the number of men and women who graduated after receiving at least one show cause $(X_1^2=0.056, \text{P}=0.813)$. There was also no significant difference in the number of UoS failed $(X_2^2=2.249, \text{P}\hspace{0.17em}=\hspace{0.17em}0.325)$ or number of show causes received $(X_6^2=2.829, \text{P}=0.830)$ between men and women.

Students who transferred from another degree program were significantly less likely to graduate $(X_1^2=13.53, \text{P}\hspace{0.17em}=\hspace{0.17em}0.0002)$. The likelihood of graduating was not statistically significant different between domestic and international students who received a show cause $(X_1^2=0.88, \text{P}<0.348)$ (Supplementary Figure S3).

### Student responses to show causes

**Fig. 3:**
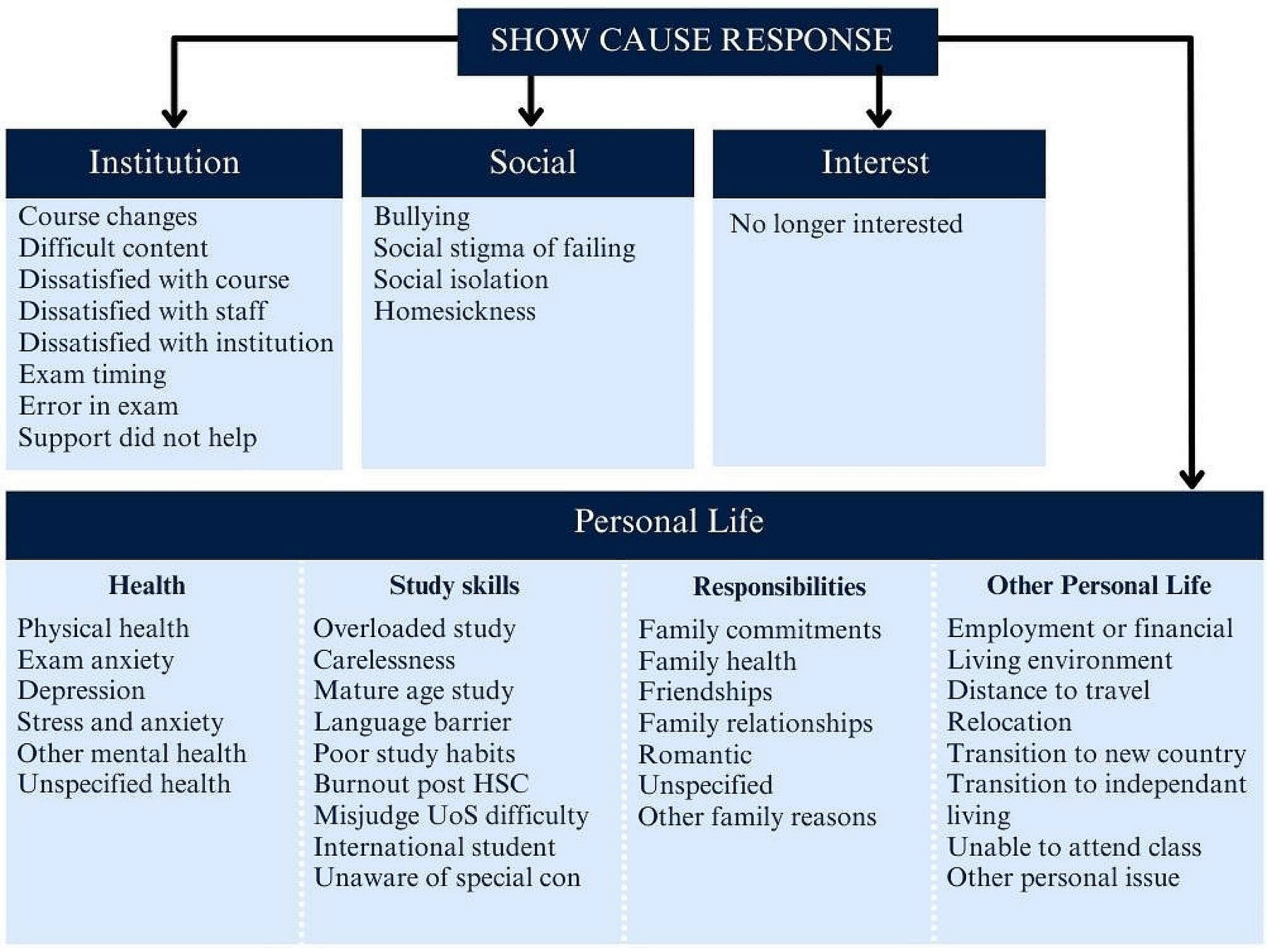
All show cause responses provided by students could be categorised into four major themes. Personal life was subcategorised into health, study skills, responsibilities, and other personal life

There were 293 show causes in total, of which only 141 show cause response letters were available. Reasons given by students for their poor performance could be classified under four major themes: personal life matters, institutional aspects, social integration, and interest in the course (Fig. 3). Personal life matters could be further sub-divided into health, study familiarity, responsibilities, and other personal life matters.

The majority of show cause responses attributed poor performance to personal life reasons (87%, 396 responses), followed by institution-related (8.8%, 40 responses), lack of interest in the degree (2.2%, 10 responses), and social integration (2%, 9 responses). The five most mentioned personal life reasons that led to poor performance were stress and anxiety (*n* = 63, 45%), physical health (*n* = 51, 36%), and depression (*n* = 39 28%). This was followed by family health, mentioned 37 times (26%), and reasons relating to employment or financial health, mentioned 33 times (23%). Reasons that related to the institution totalled 40, interest of the course totalled 10, and social reasons totalled 9. Personal life health-related reasons accounted for 41% of show cause responses. These included a combination of physical, mental, and unspecified health issues.

Some students identified a lack of study-related skills and study familiarity as a source of underperformance. Reasons included: carelessness in exams, poor study habits, language barrier, being an international student or mature age student, misjudging the course difficulty, overloading, burning out after high school, and being unaware of opportunities to apply for special consideration. Another set of reasons provided for underperformance included: needing to meet responsibilities and commitments for family, friendships, and romantic relationships. A variety of other personal life reasons were provided, which included: employment, finance, transition to independent living or a new country, living environment, distance to travel to the university, needing to relocate, and being physically unable to attend classes.

Student show cause responses that attributed poor performance to inefficiencies within the institution included UoS changes, error or poor timing of exams, dissatisfaction with the course and staff, and unhelpful support. Some students found the UoS content too difficult. Social reasons that lead to poor performance included: bullying, stigma from peers once failing, and homesickness (for those studying abroad). Another reason provided was no longer being interested or committed to the course.

## Discussion

This study investigated the key determinants of underperformance by pharmacy students at an Australian higher education institution. Our findings indicate that across the students enrolled in BPharm, BPharmMgmt, and MPharm degrees, those who had failed more UoS overall, were less likely to graduate. The types of UoS failed were weighted towards chemistry-based subjects, and the most frequent student-reported reasons for poor performance were related to personal health.

Our study also found that students who transferred from another higher education institution were less likely to graduate compared with students who had not transferred. Some studies in the US have found that students who transfer to bachelors programs from similar institutions or community colleges, which are US institutions that only offer two year undergraduate associate degrees that lead to a specific skilled job or can be used to transfer into a bachelor course [54], experience ‘transfer shock’ where grade point average (GPA) declines at the post transfer institution, which can eventually result in attrition [55, 56]. In contrast, other studies have found no significant effects from transfers, and an overall lack of consensus on this as a universal experience [57, 58]. A study that examined transferring engineering students found that students who transferred from similar degrees were more likely to graduate when compared with students who transferred from less comprehensive degrees [56]. A literature review that examined transferring student performance found factors that negatively influenced persistence and course completion included: a lack of social integration, limited transferrable credits, lower GPAs, lack of funding, distance from institution, academic rigour, and personal work/life balance [57].

Our analysis also found that students failing more than three UoS were more likely to not graduate when compared with those who failed fewer UoS. This finding parallels many studies that show students with poor academic outcomes are more likely to not complete their degree [59, 60]. A recent study on student attrition, found that students who failed one subject were more likely to fail more subjects, and also had a four-fold higher likelihood of not graduating [27]. The Grattan Institute presents similar statistics, where students who consistently fail to meet academic progression requirements eventually decide to leave or are excluded from re-enrolling by the university [61].

The high occurrence of underperformance in relation to chemistry is consistent with other studies [62, 63]. Pancyk et al. found that chemistry marks were correlated with attrition while biology marks predicted likelihood of delayed graduation for Master of Science (in Pharmacy) students. Another study found that the prior attainment of a Bachelor of Science degree to be a predictor of performance in a Doctor of Pharmacy program [64]. In countries, such as the US, where a specialised pre-admissions pharmacy test (Pharmacy College Admissions Test; PCAT) is used for entrance into a pharmacy program, the PCAT score correlated with student academic performance in the pharmacy course [65]. There are five areas examined by the PCAT, including: writing, biological processes, chemical processes, critical reading, and quantitative reasoning [66]. There is also evidence that better outcomes attained in pre-pharmacy biology and mathematics GPA [67, 68], or having completed a four-year bachelor course, contributes to student performance in American pharmacy colleges [64, 69, 70]. Another study found prior academic achievement in secondary school, or pre-university study, can predict performance in an UK MPharm course; however, not the likelihood of graduation [71]. Other studies have found that pre-tests, for certain UoS, like biochemistry and pharmaceutical calculations conducted before starting a subject are correlated with overall subject performance, which makes these tests a good predictor for at-risk students [67, 68].

The most common reasons reported by students for their underperformance in the present study were stress and anxiety, personal health, and depression. This is consistent with current literature [17, 23–27], and the 2022 Australian Student Experience Survey [72], which reported that health or stress, followed by work/life balance were the leading causes for students attrition. A specific study in pharmacy students found that exam anxiety had a negative impact on student performance in pharmacy practical exams [26]. Psychological distress among students completing a higher education degree in Norway showed negative impacts on their self-perceived academic ability, and course progression [73]. Another study investigating students’ self-reported explanations for their poor academic performance found mental health as a contributing factor, and vice versa, where poor performance intensified mental distress [27]. Although the Australian Bureau of Statistics also reported personal health reasons as a major contributor for non-completion in bachelor programs between 2018 and 2019, the leading reason was that students were no longer interested in their chosen degree. In the same report, non-completion of masters degrees was driven by family, health, or other personal reasons [42]. Student mental health is a significant driver of attrition and is common across both private and public higher institutions in Australia [41]. The mental health burden on students is recognised at The University of Sydney and so significant mental health support is offered. All students are able to access free counselling and psychological support sessions, there is a 24/7 mental health support telephone line, and additional self-help resources (like mindfulness and relaxation) are provided through the university’s website. Mental health first health training is also included in the curricula for all pharmacy degree programs at the university.

Successful completion of a pharmacy degree requires not only academic ability, but a certain level of pre-knowledge, in particular, biology and chemistry, to decrease failure rates in these subjects, avoid delays in degree completion, and possible attrition. Institutions should aim to address these barriers by introducing pre-requisite subjects or mandate compulsory bridging courses if a prior level of knowledge attainment in these subject areas is not provided. Alternatively, pre-tests for certain UoS can be conducted prior to the course commencement to identify at-risk students, and additional academic support services can be offered.

With student poor mental health found as the most common self-reported reason for poor performance in this study, often exacerbated by academic performance pressures, institutions should implement policies for early detection and support for students going through challenging times. Such policies could include more frequent reminders for students to self-assess their mental health, and information on where to seek support services. This could take form in programs being introduced prior to lectures, access to support portals made more prominent on online learning platforms, or self-check surveys to be taken at a frequency deemed appropriate.

### Limitations

The present study had a number of limitation. Not all student’s ATAR scores (or equivalent) were available. The method of collecting whether a student was registered as a pharmacist was based on them not having changed their last name which may be the case for some students who changed their name after graduation (e.g. upon marriage). Students who may be registered as a pharmacist in countries other than Australia could not be determined. Not all student show cause reasons were available because of the change from physical to electronic filing over the period studied. The limited number of students who received five or more show causes also meant the study was not powered to establish a cut-off whereby after receiving a certain number of show causes, the chance of graduating is highly unlikely.

## Conclusions

This study investigated the key determinants for poor academic performance in a cohort of pharmacy students enrolled in a BPharm, BPharmMgmt, and MPharm degree. The key factors that influenced whether a show cause student completed their studies included whether they transferred from another institution, and failed more than three UoS. The UoS with the highest fail rates were chemistry based, and the most frequent student self-reported reason for poor performance was personal stress and anxiety. The results indicate that pharmacy schools should aim to address student foundation knowledge in chemistry, identify at-risk students early using pre-subject testing, and provide better access and knowledge of available services to address student mental burden. Future studies should investigate whether students who have completed chemistry and biology pre-requisites perform better in their pharmacy degree.

### Electronic supplementary material

Below is the link to the electronic supplementary material.

Supplementary Material 1

## Data Availability

The data that support the findings of this study are available on request from the corresponding author, N.J.W.
