# Supplementary material for: Causes and outcomes of at-risk underperforming pharmacy students: implications for policy and practice
Source: BMC Med Educ. 2024 Apr 19;24:421. doi: 10.1186/s12909-024-05327-z (PMC11027423; doi:10.1186/s12909-024-05327-z)
Supplement: Supplementary file 1 — Supplementary Material 1 [file 12909_2024_5327_MOESM1_ESM.docx]

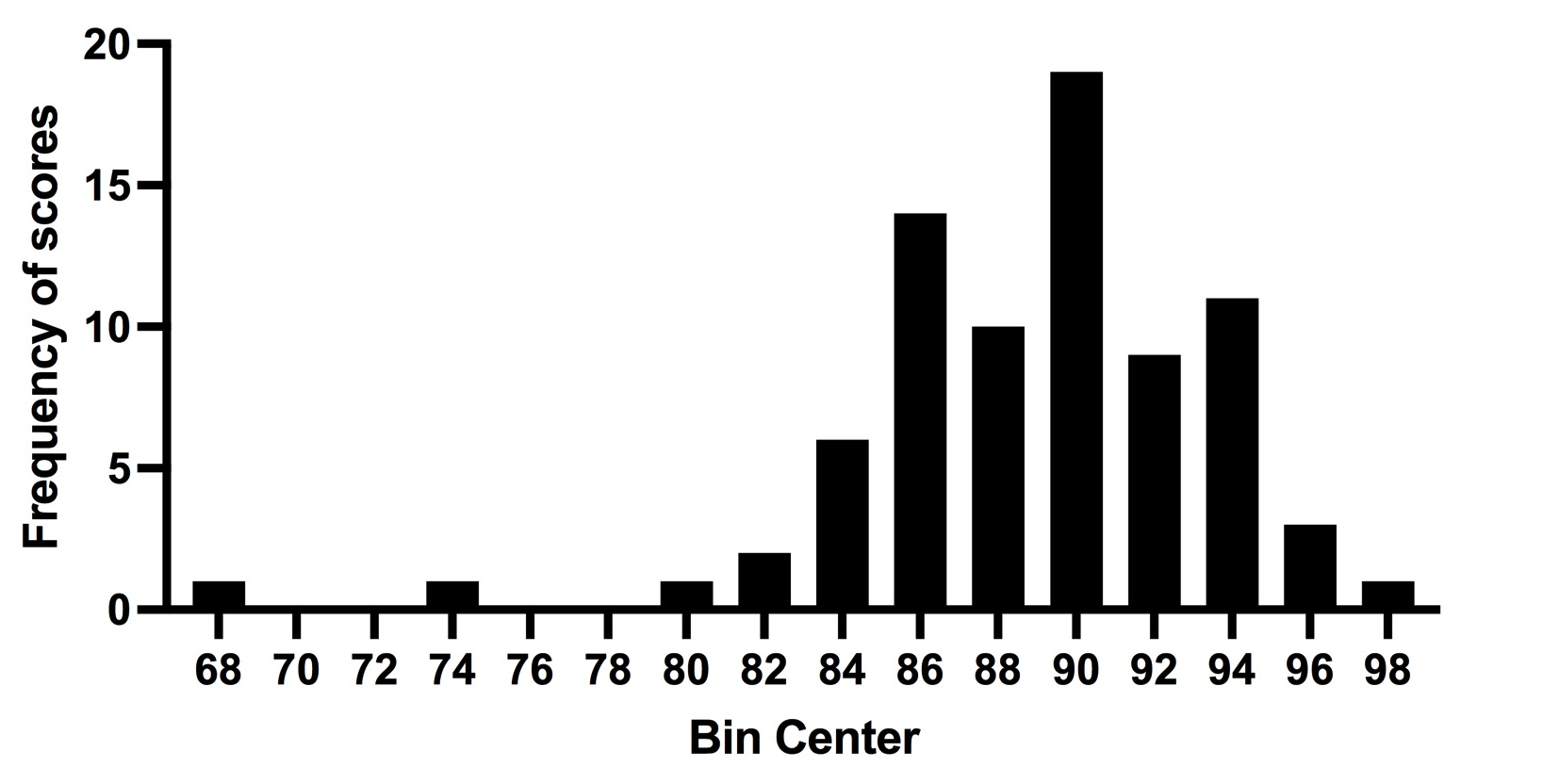


**ATAR**

**Figure S1.** The ATAR score of students in the BPharm or BPharm/Mgmt degrees who received at least one show cause over the duration of their course

**Table S1:** Proportion of students who graduated across ATAR bands. Data are expressed as a proportion of the total number of students within each ATAR band.

| **ATAR** | **Proportion of students graduating**  **% (n)** |
| --- | --- |
| <85 | 27.3 (11) |
| 85-89.99 | 44.1 (34) |
| 90-94.99 | 34.5 (29) |
| 95+ | 25.0 (4) |


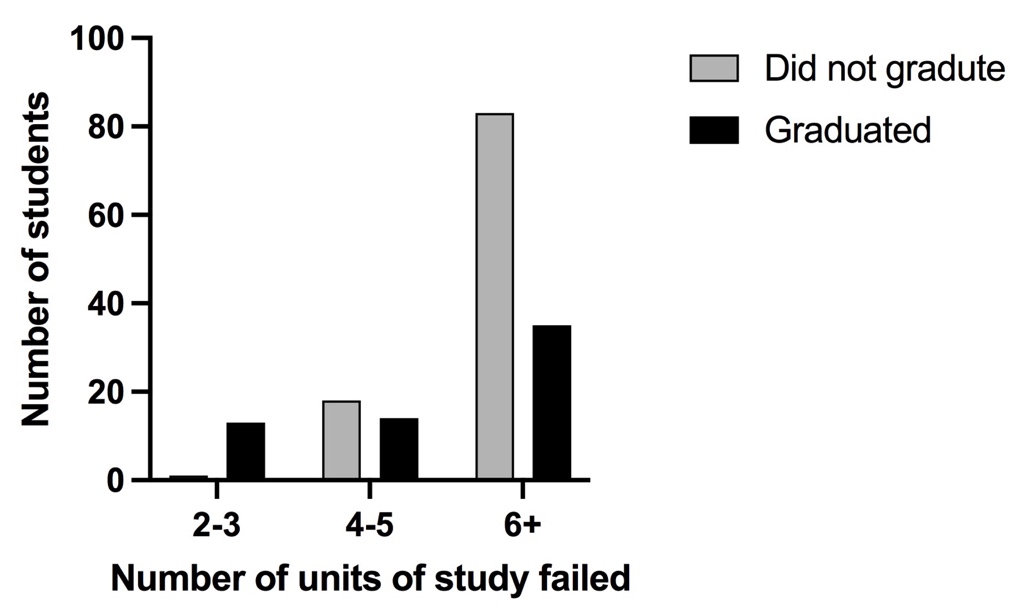


**Figure S2.** Number of students who graduated (black) and did not graduate (grey) as a function of the number of UoS failed.


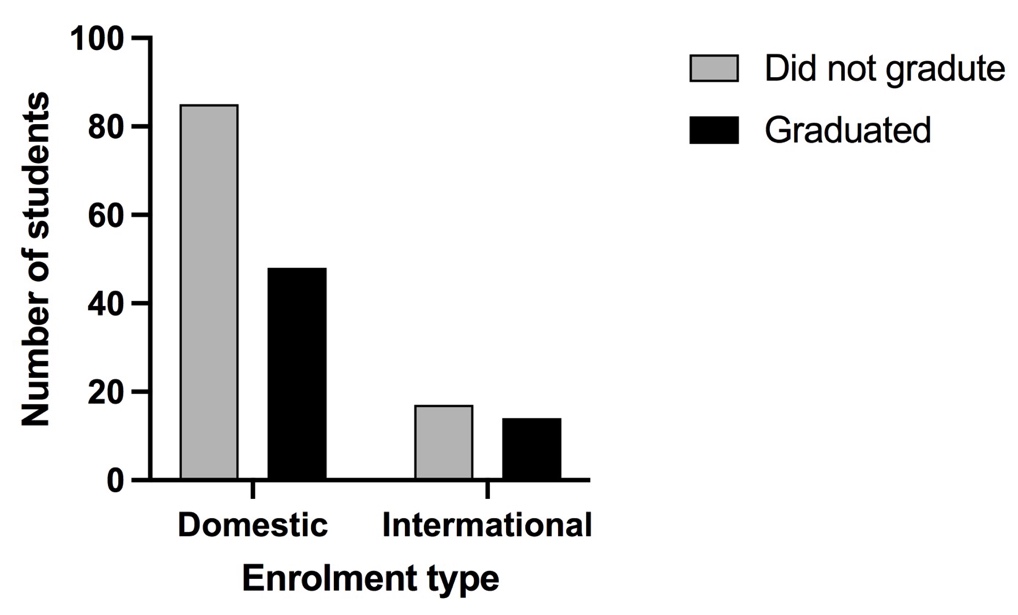


**Figure S3:** Domestic vs international students that graduated (black) and did not graduate (grey) after receiving a show cause.
